# The Fusion of *CLEC12A* and *MIR223HG* Arises from a *trans*-Splicing Event in Normal and Transformed Human Cells

**DOI:** 10.3390/ijms222212178

**Published:** 2021-11-10

**Authors:** Bijay P. Dhungel, Geoffray Monteuuis, Caroline Giardina, Mehdi S. Tabar, Yue Feng, Cynthia Metierre, Sarah Ho, Rajini Nagarajah, Angela R. M. Fontaine, Jaynish S. Shah, Divya Gokal, Charles G. Bailey, Ulf Schmitz, John E. J. Rasko

**Affiliations:** 1 Gene and Stem Cell Therapy Program Centenary Institute, The University of Sydney, Camperdown, NSW 2050, Australia; b.dhungel@centenary.org.au (B.P.D.); c.giardina@centenary.org.au (C.G.); m.tabar@centenary.org.au (M.S.T.); j.feng@centenary.org.au (Y.F.); C.Metierre@centenary.org.au (C.M.); s.ho@centenary.org.au (S.H.); r.nagarajah@centenary.org.au (R.N.); j.shah@centenary.org.au (J.S.S.); d.gokal@centenary.org.au (D.G.); c.bailey@centenary.org.au (C.G.B.); 2 Faculty of Medicine & Health, The University of Sydney, Camperdown, NSW 2006, Australia; 3 Department of Biochemistry and Developmental Biology, University of Helsinki, 00014 Helsinki, Finland; geoffray.monteuuis@helsinki.fi; 4 Centenary Imaging and Sydney Cytometry Centenary Institute, The University of Sydney, Camperdown, NSW 2050, Australia; a.fontaine@centenary.org.au; 5 Cancer & Gene Regulation Laboratory Centenary Institute, The University of Sydney, Camperdown, NSW 2050, Australia; 6 Department of Molecular & Cell Biology, College of Public Health, Medical & Vet Sciences, James Cook University, Townsville, QLD 4811, Australia; 7 Cell and Molecular Therapies, Royal Prince Alfred Hospital, Camperdown, NSW 2050, Australia

**Keywords:** chimeric RNAs, Fusion RNAs encoding protein, fusion transcript, linc-223, miR-223 host gene, trans-splicing, alternative splicing, CCL1, myeloid cell differentiation, C-type lectin, chronic myeloid leukemia

## Abstract

Chimeric RNAs are often associated with chromosomal rearrangements in cancer. In addition, they are also widely detected in normal tissues, contributing to transcriptomic complexity. Despite their prevalence, little is known about the characteristics and functions of chimeric RNAs. Here, we examine the genetic structure and biological roles of *CLEC12A-MIR223HG*, a novel chimeric transcript produced by the fusion of the cell surface receptor *CLEC12A* and the *miRNA-223* host gene (*MIR223HG*), first identified in chronic myeloid leukemia (CML) patients. Surprisingly, we observed that *CLEC12A-MIR223HG* is not just expressed in CML, but also in a variety of normal tissues and cell lines. *CLEC12A-MIR223HG* expression is elevated in pro-monocytic cells resistant to chemotherapy and during monocyte-to-macrophage differentiation. We observed that *CLEC12A-MIR223HG* is a product of *trans*-splicing rather than a chromosomal rearrangement and that transcriptional activation of *CLEC12A* with the CRISPR/Cas9 Synergistic Activation Mediator (SAM) system increases *CLEC12A-MIR223HG* expression. *CLEC12A-MIR223HG* translates into a chimeric protein, which largely resembles CLEC12A but harbours an altered C-type lectin domain altering key disulphide bonds. These alterations result in differences in post-translational modifications, cellular localization, and protein–protein interactions. Taken together, our observations support a possible involvement of *CLEC12A-MIR223HG* in the regulation of *CLEC12A* function. Our workflow also serves as a template to study other uncharacterized chimeric RNAs.

## 1. Introduction

Chimeric RNAs are transcripts that consist of exons from different parental genes. They can be produced by several mechanisms which may or may not involve chromosomal translocations at the genomic level. Transcription of fusion genes resulting from chromosomal deletion, inversion, or translocation are considered to be a hallmark of cancer [1,2]. These fusion genes can give rise to proteins with important roles in cancer development and progression and may also serve as therapeutic targets. Some examples include the BCR-ABL1 fusion protein in chronic myeloid leukaemia (CML) [3,4] and EML4-ALK in lung cancer [5].

Apart from chimeric RNAs produced by DNA-level gene fusions, studies have uncovered additional mechanisms that are known to produce chimeric transcripts at the RNA level [1,6,7,8]. These include intergenic splicing by either *cis*-splicing, which involves same-strand neighbouring genes or by *trans*-splicing, whereby exons from two separate RNA transcripts are spliced together [1,9,10]. Although *cis*- and *trans*- splicing events were originally thought to be rare in mammals, high-throughput analyses of transcriptomes have revealed that significant portions of chimeric RNAs are derived from intergenic splicing [11,12,13].

The expression of chimeric RNAs is significantly higher in cancer compared to normal tissues [1,14,15]. As such, they may serve as potential biomarkers for diagnosis, prognosis or even therapeutic targets. However, the detection of chimeric RNAs in non-malignant tissues has led to investigations of their roles in normal physiology and development [6,16]. Chimeric RNAs could be fundamental to basic cellular processes and provide a means for expansion of the functional genome, i.e., transcriptomic complexity, without an increase in the number of genes [16,17]. For example, *SLC45A3-ELK4* is a recurrent *cis*-spliced chimeric transcript expressed in normal prostate as well as prostate cancer to modulate cell proliferation [18]. *JAZF1-SUZ12* is a protein-coding chimeric transcript produced by a physiologically-regulated *trans*-splicing mechanism, which impairs chromatin repression [19,20]. During normal myogenesis, *PAX3-FOXO1*, a chimeric RNA, is detected transiently without any evidence of chromosomal rearrangement [21] and interferes with the TGF-β pathway [22].

Technological and analytical advancements made in the past decade offer unique opportunities to appreciate the prevalence and explore the relevance of chimeric RNAs in normal physiology and cancer [10,14,16]. While they are now widely recognised as being ubiquitous, chimeric RNAs are largely under-investigated, and their understanding is limited. Currently, most studies use bioinformatics pipelines to search for chimeric RNAs in RNA sequencing data from different biological sources [23,24,25,26]. These studies have identified thousands of chimeric transcripts in both malignant and normal tissues [27]. However, very few have been experimentally characterized in detail.

In a recent study, we used RNA sequencing to study the changing transcriptomes in CML patients [28]. In addition to striking changes in alternatively spliced transcripts, we identified known and novel fusion transcripts. In the present study, we analysed the most frequently recurring uncharacterized fusion transcript in CML: namely *CLEC12A-MIR223HG*. This chimeric transcript is produced by the fusion of *CLEC12A*, a myeloid-inhibitory receptor and the host gene of myeloid regulatory miRNA-223 (*MIR223HG*). We examined the genetic architecture and the potential fusion protein encoded by the *CLEC12A-MIR223HG* chimeric transcript. We have also conducted a range of cell biological assays to investigate the function of *CLEC12A-MIR223HG* and its putative role in cancer biology. Our results suggest a possible role of *CLEC12A-MIR223HG* in the regulation of *CLEC12A* function. These results also provide a template for further investigations into the biological significance of *CLEC12A-MIR223HG* and other chimeric RNAs.

## 2. Results

### 2.1. CLEC12A-MIR223HG Is Expressed in CML Patients and Healthy Controls

**Figure 1:**
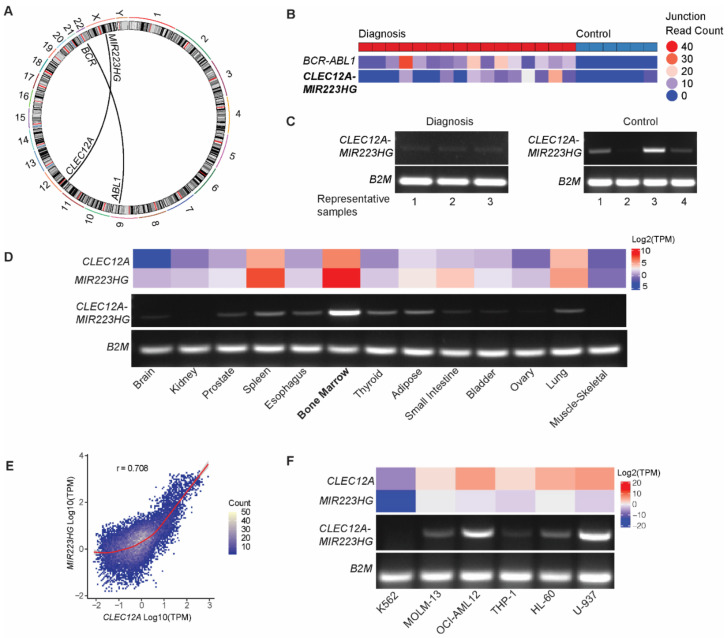
*CLEC12A-MIR223HG* detection in CML patients, controls, normal tissues, and cell lines. (**A**) Reads mapping across the *CLEC12A/MIR223HG* breakpoint. (**B**) Read counts that span across the fusion breakpoint in representative CML diagnostic and control samples. (**C**) RT-PCR-based detection of *CLEC12A-MIR223HG* in CML diagnostic and control samples with primers flanking the breakpoint. Amplicon sizes expected: *B2M*: 96 bp, *CLEC12A-MIR223HG*: 207 bp. (**D**) RT-PCR-based detection of *CLEC12A-MIR223HG* in diverse normal tissues and RNA sequencing-based quantification of *CLEC12A* and *MIR223HG* transcript levels. (**E**) Contour plot illustrating correlation between *CLEC12A* and *MIR223HG* across different tissues. Correlation coefficient (r) was determined using Pearson correlation and the red line indicates the line of best fit. (**F**) RT-PCR-based detection of *CLEC12A-MIR223HG* in leukemic cell lines. TPM (transcripts per million) values for tissues were obtained from the Genotype-Tissue Expression (GTEx) Portal V8 (https://gtexportal.org) (accessed on 25 October 2021). TPM values for cell lines were obtained from the Cancer Cell Line Encyclopedia project portal (https://depmap.org/portal/ccle/) (accessed on 25 October 2021).

We have previously reported next generation sequencing-based gene fusion analysis on poly(A)-enriched RNA from peripheral blood mononuclear cells (PBMCs) of 16 Philadelphia-positive (Ph+) CML patients at diagnosis and ten matched remission samples from the same individuals, as well as six healthy control subjects [28]. All the CML patients were treated with tyrosine kinase inhibitors (TKIs). Sequencing reads mapping the *BCR-ABL1* locus served as a control. Most frequently recurring, apart from the characteristic *BCR-ABL1* fusion, was *CLEC12A-MIR223HG*, which is a fusion transcript between the transmembrane glycoprotein *CLEC12A* (a.k.a. *CCL1*) and the *miR223* host gene *MIR223HG*, located at chromosomal loci 12p13.31 and Xq12, respectively. Interestingly, we observed nine distinct fusion events involving *CLEC12A*, which encodes a myeloid-inhibitory C-type lectin receptor known to play an important role in haematopoiesis [29]. Seven out of the nine breakpoints were identical (Supplementary Table S1). *CLEC12A* is also a cancer stem cell marker in myelodysplastic syndrome [30] and a therapeutic target in acute myeloid leukemia [31]. Based on its high recurrence frequency and interesting biology associated with both the parental genes, *CLEC12A-MIR223HG* was chosen for further examination (Figure 1A).

*CLEC12A-MIR223HG* was detected in 10/16 diagnosis samples, 0/10 of the remission samples, and in 1/6 of healthy control samples. While the fusion breakpoints were identical among *CLEC12A-MIR223HG*-positive samples (left: chr12:9982129; right: chrX: 66020127), read counts across the breakpoints differed, indicating varying expression levels of the chimeric transcript (Figure 1B). Surprisingly, using RT-PCR, the *CLEC12A-MIR223HG* fusion could also be detected at low levels in normal control samples (Figure 1C). These results indicate that despite being expressed at CML diagnosis, *CLEC12A-MIR223HG* cannot serve as a prognostic marker for TKI-based therapies.

### 2.2. CLEC12A-MIR223HG Is Expressed in Normal Human Tissues and Leukemic Cell Lines

As *CLEC12A-MIR223HG* is not specific to CML pathology, we sought to examine its expression in different tissues. As indicated in Figure 1D, the expression of *CLEC12A-MIR223HG* is highest in the bone marrow, where both *CLEC12A* and *MIR223HG* are also individually expressed at high levels. Using Pearson correlation, a strong correlation between the levels of *CLEC12A* and *MIR223HG* (r = 0.708) across 30 different tissues was observed (Figure 1E). We also observed tissue-specific differences in the correlation strength (Figure S1). Next, we studied the expression of *CLEC12A-MIR223HG* in different leukemia cell lines. As depicted in Figure 1F, *CLEC12A-MIR223HG* is expressed at varying levels in several leukemic cell lines. Due to a high expression of *CLEC12A-MIR223HG*, U-937 pro-monocytic cells were used for further characterization.

### 2.3. CLEC12A-MIR223HG Exhibits a Typical Fusion Transcript Architecture

**Figure 2:**
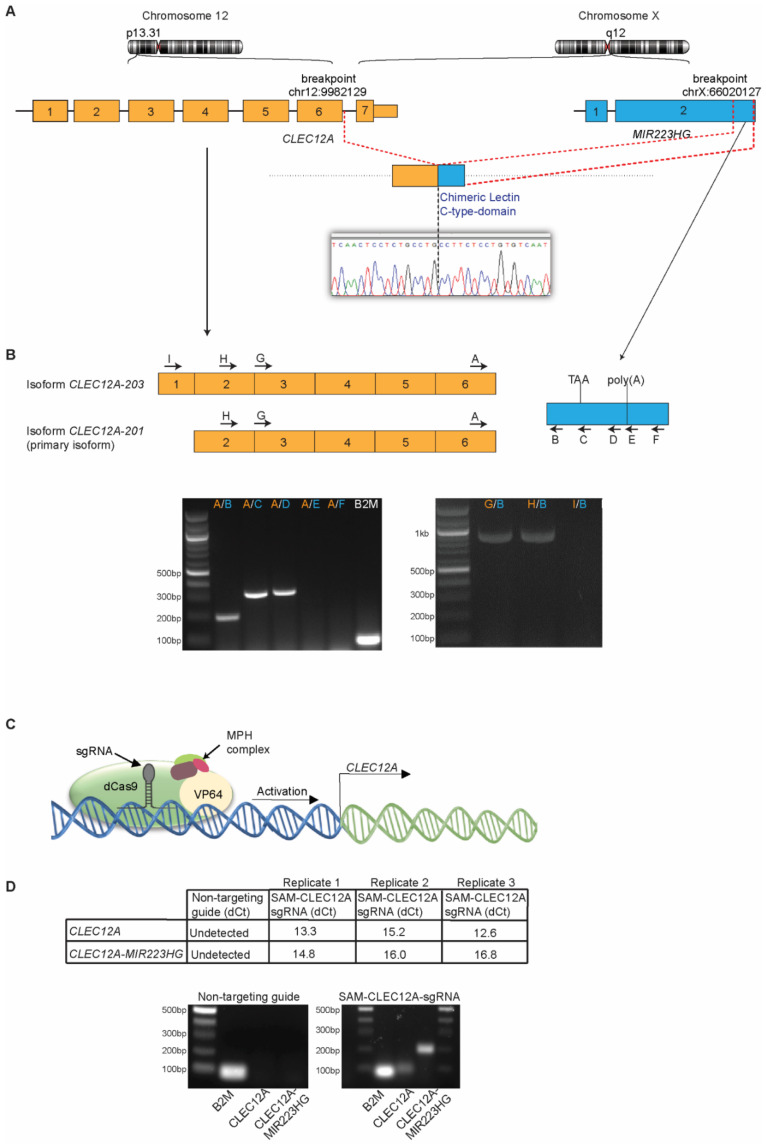
Transcript structure of *CLEC12A-MIR223HG*. (**A**) Illustration of the breakpoint location and the resulting fusion transcript. (**B**) RT-PCR on U-937 mRNA to identify the full length *CLEC12A-MIR223HG* transcript. Expected amplicon sizes are as follows: A/B: 207 bp, A/C: 361 bp, A/D: 371 bp, A/E: 564 bp, A/F: 579 bp, H/B: 829 bp, G/B: 962 bp. The chimeric transcript is produced by the substitution of the final exon of *CLEC12A-201* isoform with a part of *MIR223HG* including an in-frame stop codon followed by a poly(A) sequence. (**C**) Illustration of the CRISPR/SAM-based transcriptional activation system used to increase the endogenous expression of *CLEC12A*. (**D**) Three independent replicates of qRT-PCR accessing expression levels of *CLEC12A* and *CLEC12A-MIR223HG* after CRISPR/SAM-mediated transcriptional activation of *CLEC12A* in HEK293 cells. Expected amplicon sizes are as follows: *B2M*: 91 bp, *CLEC12A*: 105 bp, *CLEC12A-MIR223HG*: 206 bp. B2M was used to determine the dCt values.

To determine the architecture of the *CLEC12A-MIR223HG* transcript, we performed RT-PCR with primers specific to different isoforms of *CLEC12A* (Figure 2A,B). As *MIR223HG* is polyadenylated, we sought to determine if *CLEC12A-MIR223HG* also possesses a poly(A)-tail by designing primers around the canonical poly(A) signal of *MIR223HG*. RT-PCR analysis indicates that *CLEC12A-MIR223HG* harbours the poly(A) signal from *MIR223HG* (Figure 2B). Furthermore, as we used oligo(dT) as primer for cDNA synthesis, it is likely that *CLEC12A-MIR223HG* is polyadenylated. Next, we investigated the transcription start site of *CLEC12A-MIR223HG* by identifying the transcript isoform of *CLEC12A* that engages in the fusion event. As indicated by the RT-PCR results (Figure 2B), the primary transcript isoform of *CLEC12A* (*CLEC12A*-201) participates in the fusion.

### 2.4. CRISPR-Mediated Transcriptional Activation of CLEC12A Increases CLEC12A-MIR223HG Expression

As *CLEC12A* and *MIR223HG* are located on different chromosomes, *CLEC12A-MIR223HG* could theoretically be produced either by chromosomal rearrangement or *trans*-splicing. To distinguish these alternatives, we used HEK293 cells, which do not express detectable levels of either *CLEC12A* or *CLEC12A-MIR223HG* by RT-qPCR (data not shown). We searched for all the detectable chromosomal translocations in HEK293 cells but found no translocations between chromosome 12 and chromosome X (Supplementary Table S4). Next, we exploited the CRISPR/Cas9 Synergistic Activation Mediator (CRISPR/SAM)-based transcriptional activation technology to further examine the mechanisms regulating *CLEC12A-MIR223HG* expression. We engineered HEK293 cells to stably express the components of the CRISPR activation system (dCas9/MPH) and transduced these cells with a lentivirus that expresses a short guide RNA (sgRNA) that targets the *CLEC12A* promoter region (Figure 2C). Only a sgRNA targeting the *CLEC12A* promoter induced the expression of *CLEC12A-MIR223HG* to levels detectable with qRT-PCR (Figure 2D). Furthermore, *CLEC12A-MIR223HG* was only amplified from the cDNA and not the genomic DNA of U-937 cells (Figure S2E). When combined, these results suggest that *CLEC12A-MIR223HG* is likely to be the result of *trans*-splicing rather than a consequence of chromosomal rearrangement.

### 2.5. CLEC12A-MIR223HG Encodes a Chimeric Protein That Is Distinct from CLEC12A

**Figure 3:**
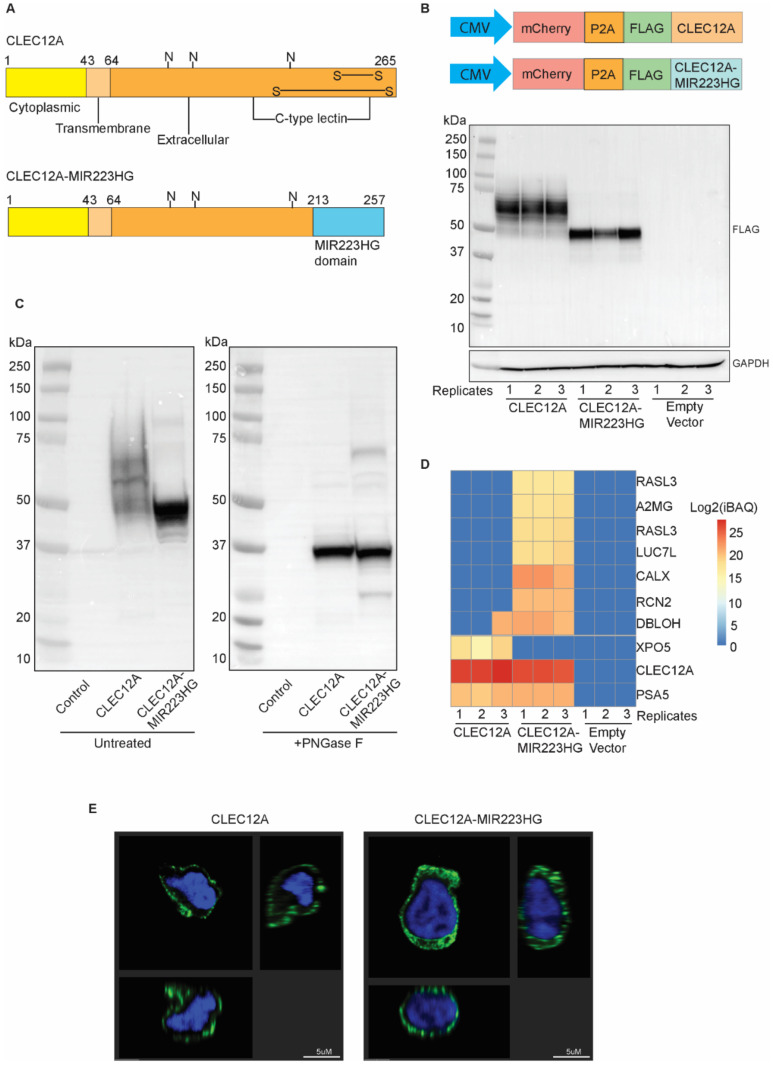
*CLEC12A-MIR223HG* encodes a chimeric protein distinct from CLEC12A. (**A**) Schematic of CLEC12A and CLEC12A-MIR223HG protein architectures. (**B**) Western blot image of cell lysates from HEK293 cells transduced with lentiviral vectors expressing either *CLEC12A* or *CLEC12A-MIR223HG*. (**C**) Western blot image of PNGase F-treated and untreated U-937 cell lysates overexpressing *CLEC12A* or *CLEC12A-MIR223HG*. (**D**) Immunoprecipitation with anti-FLAG antibody coupled with mass spectrometry of U-937 cells expressing either *CLEC12A* or *CLEC12A-MIR223HG*. Total peptide intensity divided by the number of observable peptides for a particular protein (iBAQ) is depicted. Compared to CLEC12A, the chimeric protein has gained interacting partners and lost at least one. Three separate replicates were performed. (**E**) Representative images of immunofluorescence with anti-FLAG antibody (green) and DAPI (blue) of U-937 cells expressing either *CLEC12A* or *CLEC12A-MIR223HG*. Image analysis was performed using Biplane Imaris software. Three different angels of the same cell are depicted.

Next, we investigated whether *CLEC12A-MIR223HG* translates into a chimeric protein. The chimeric RNA transcript sequence determined by RT-PCR and Sanger sequencing enabled us to analyse the potential protein encoded by *CLEC12A-MIR223HG*. Our analysis indicated that CLEC12A-MIR223HG results in a substitution of the last 52 amino acids (aa 214–265) of CLEC12A with 44 new amino acids and a new 3′ UTR from *MIR223HG*. This would result in a chimeric protein containing the cytoplasmic and transmembrane domain of CLEC12A. However, the extracellular domain, which contains the C-type lectin, would be altered so as to disrupt key disulfide bonds (Figure 3A).

Due to the lack of a suitable antibody specific to the chimeric domain of the fusion protein, we cloned the coding sequences of both *CLEC12A* and *CLEC12A-MIR223HG* into a lentiviral expression vector and N-terminally tagged them with a FLAG epitope. After overexpressing *CLEC12A* in different cell lines, we detected FLAG-tagged CLEC12A ranging between 40 and 75 kDa (Figure 3B and Figure S2A). In contrast, ectopic expression of the *CLEC12A-MIR223HG* coding sequence produced a single prominent band between 37 and 50 kDa (higher than the predicted size of 37 kDa) (Figure 3B). To determine whether the observed higher molecular weight of CLEC12A-MIR223HG was due to glycosylation as previously described [32], U-937 cell lysates overexpressing either *CLEC12A* or *CLEC12A-MIR223HG* were treated with PNGase F to remove N-linked glycosylation and examined by Western blotting (Figure 3C). A predominant band was observed at ~37 kDa for both proteins (Figure 3C), suggesting that CLEC12A and CLEC12A-MIR223HG undergo distinct glycosylation programs. Both proteins have three predicted N-glycosylation sites, but the chimeric CLEC12A-MIR223HG could disrupt normal post-translational modifications of CLEC12A (Figure 3A).

To investigate the fusion protein further, we performed immunoprecipitation coupled with mass spectrometry and identified interacting partners of CLEC12A and CLEC12A-MIR223HG. We identified interactors unique to CLEC12A-MIR223HG, which included CALX, RHG04, A2MG, RCN2, RASL3, and LUC7L. PSA5 was detected in pulldowns of both CLEC12A and CLEC12A-MIR223HG (Figure 3D). A unique peptide sequence originating from the MIR223HG side of the fusion protein (HDLGNCPR) confirmed its expression. These results suggest that the chimeric lectin domain in CLEC12A-MIR223HG facilitates novel protein interactions.

Next, we performed immunofluorescence on U-937 overexpressing FLAG-tagged-CLEC12A or -CLEC12A-MIR223HG (Figure 3E). Similar to CLEC12A, CLEC12A-MIR223HG localised to the plasma membrane. However, the chimeric protein was also detected in the cytoplasmic compartment like most of its interacting partners (Figure 3E and Supplementary Table S2). When combined, these results suggest that CLEC12A-MIR223HG is a chimeric protein differing from CLEC12A in its patterns of post-translational modifications, interactions with other proteins, and sub-cellular localization.

### 2.6. Increased CLEC12A-MIR223HG Expression following Cell Differentiation or Chemotherapy

**Figure 4:**
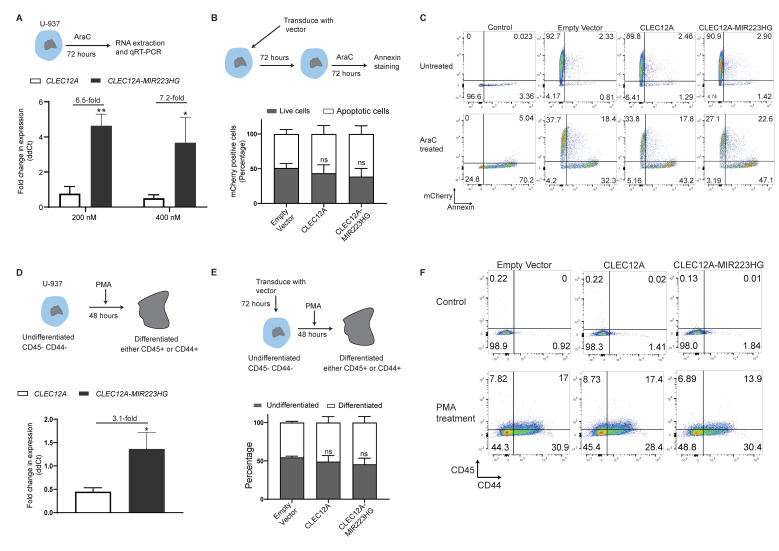
Biological roles of *CLEC12A-MIR223HG*. (**A**) Fold change in the expression of *CLEC12A* and *CLEC12A-MIR223HG* in U-937 cells treated with two different doses of cytarabine (AraC) measured by qRT-PCR. (**B**) Percentage of apoptotic cells (Annexin V-positive cells) after the treatment of U-937 cells overexpressing *CLEC12A* or *CLEC12A-MIR223HG* with cytarabine. (**C**) Representative flow cytometry plots depicting percentage of apoptotic U-937 cells overexpressing either *CLEC12A* or *CLEC12A-MIR223HG* after AraC treatment. *X*-axis: Annexin V, *Y*-axis: mCherry (depicting transduction efficiency). (**D**) Fold change in the expression of *CLEC12A* and *CLEC12A-MIR223HG* during the differentiation of U-937 monocytes induced by phorbol 12-myristate 13-acetate (PMA) treatment (measured by qRT-PCR). (**E**) Percentage of either CD45+ or CD44+ cells after the treatment of U-937 cells overexpressing *CLEC12A* or *CLEC12A-MIR223HG* with PMA. (**F**) Representative flow cytometry plots depicting the percentage of either or CD44 (*x*-axis) or CD45 (*y*-axis) in PMA-treated U-937 monocytes overexpressing *CLEC12A* or *CLEC12A-MIR223HG* (*n* > 3, * *p* < 0.05, ** *p* < 0.01).

To investigate possible functions of *CLEC12A-MIR223HG* in cancer and normal biology, we performed a range of cell biology assays. We did not observe a significant impact of either *CLEC12A* or *CLEC12A-MIR223HG* overexpression in the proliferation (MTT assay) or survival (clonogenicity assay) of U-937 and THP1 cell lines (Figure S2B). We examined whether *CLEC12A-MIR223HG* could alter chemosensitivity. We treated U-937 cells with cytarabine (AraC; 200 nM) (Figure S2C,D) and observed a 6.5-fold increase in *CLEC12A-MIR223HG* expression when compared to *CLEC12A* (*p* < 0.01, Figure 4A). Similarly, treatment with 400 nM AraC resulted in a 7.2-fold increase (*p* < 0.05, Figure 4A). However, upon overexpression, neither *CLEC12A* nor *CLEC12A-MIR223HG* conferred either resistance or sensitivity to AraC (Figure 4B,C).

As *CLEC12A* expression decreases upon differentiation of monocytes, we examined whether *CLEC12A-MIR223HG* follows a similar pattern. We treated U-937 pro-monocytic cells with phorbol 12-myristate-13-acetate (PMA) to induce their differentiation. As depicted in Figure 4D, we observed a 3.1-fold increase in *CLEC12A-MIR223HG* expression compared to *CLEC12A* (*p* < 0.05). Next, we tested whether the overexpression of *CLEC12A* or *CLEC12A-MIR223HG* could alter the degree of differentiation of U-937 cells. Interestingly, no significant differences in the differentiation of U-937 were observed after the overexpression of either *CLEC12A* or *CLEC12A-MIR223HG* (Figure 4E,F). These results suggest that despite being upregulated during cell differentiation and chemotherapy, *CLEC12A-MIR223HG* does not drive these processes.

## 3. Discussion

Gene expression is a finely tuned process, which is tightly regulated by many layers of regulatory mechanisms. Regulation of gene expression can occur at transcriptional, post-transcriptional, translational, and post-translational levels. Chimeric RNAs are produced by the fusion of different parental transcripts and are now widely recognised as an additional layer of transcriptomic complexity. Some fusion transcripts arising from chromosomal rearrangements in cancer like *BCR-ABL1* are well characterized and have significant biological functions [4]. Many other fusion transcripts await further study. In our published investigation, we compared the transcriptomic landscapes of healthy donors and CML patients at diagnosis and remission [28]. We observed a number of fusion transcripts involving *CLEC12A*. In this study, we examined the most frequently recurring fusion transcript, *CLEC12A-MIR223HG,* which results from the fusion between *CLEC12A* and the *miR-223* host gene *MIR223HG*.

*CLEC12A* is located on chromosome 12 and encodes a type II transmembrane glycoprotein. It is a myeloid cell-inhibitory receptor that can recognize uric acid crystals to alert the immune system of cell death and consequently inhibit an inflammatory response [33]. The *MIR223HG* gene is located on chromosome X [34] and is a key regulator of myeloid cell differentiation [35,36]. We observed a higher expression of *CLEC12A-MIR223HG* in CML diagnostic patient samples compared to remission and control samples. Consistent with similar reports of other chimeric transcripts [12,37], the lower expression of *CLEC12A-MIR223HG* was concurrent with reduced expression of the parental transcripts. We then exploited the CRISPR-based transcriptional activation system to investigate whether *CLEC12A-MIR223HG* results from a *trans*-splicing event. We activated the endogenous expression of *CLEC12A* in HEK293 cells that do not express detectable levels of either *CLEC12A* or *MIR223HG* and do not have a chromosomal translocation between chromosome 12 and chromosome X. Both *CLEC12A* and *CLEC12A-MIR223HG* were detected after transcriptional activation of *CLEC12A*. These observations suggested that a chromosomal translocation is not essential for the production of the chimeric *CLEC12A-MIR223HG* transcript and that it arises from a *trans*-splicing event.

We examined the expression of *CLEC12A-MIR223HG* in a range of normal tissue types and cell lines. As *CLEC12A-MIR223HG* was detected in several healthy controls, it could not serve as a prognostic marker for TKI-based therapies. As expected, the expression of *CLEC12A-MIR223HG* was highest in blood, which also has high expression levels of both the parental genes. Our results mirror other studies that first identified fusion transcripts in cancer but detected their expression in normal cells upon subsequent examination [12,38]. Additionally, the expression of *CLEC12A-MIR223HG* is similar to other chimeric RNAs expressed in normal tissues that are increased in cancer [6,39].

The CLEC12A-MIR223HG fusion protein results in a substitution of 52 amino acids of the C-type lectin domain of CLEC12A with 44 amino acids arising from the DNA sequence of the *MIR223HG* taking part in the fusion. As the C-type lectin domain of CLEC12A is important for its function [33,40,41], it was not surprising that CLEC12A-MIR223HG showed striking differences with CLEC12A in terms of post-translational modifications, cellular localization, and protein–protein interactions. We noted that the molecular weights of both CLEC12A and CLEC12A-MIR223HG were higher than predicted in Western blots. It was previously reported that this could be a result of N-glycosylation of CLEC12A [32]. While CLEC12A showed multiple bands between 37 and 50kDa, a single band was observed for CLEC12A-MIR223HG. This was surprising because CLEC12A-MIR223HG retains all predicted N-glycosylation sites of CLEC12A. Consistent with a previous report [32], the treatment of cell lysates with PNGase F to remove N-glycosylation, resulted in bands at predicted molecular weights for both CLEC12A and CLEC12A-MIR223HG. We also observed a difference in interacting partners and cellular localizations of CLEC12A and CLEC12A-MIR223HG. Based on previous reports of other receptor proteins, we hypothesize that the change in the extracellular domain of CLEC12A either affects its cell surface trafficking or its stability at the cell surface [42,43]. Furthermore, post-translational modifications like glycosylation can also impact the cell surface expression of a protein [44]. It is also of note that the expression of the native fusion protein in tissues remains to be examined. These results suggest that the substitution of 52 amino acids in CLEC12A with 44 amino acids from MIR223HG results in a fusion protein with an altered glycosylation, sub-cellular localization, protein–protein interactions, possibly leading to different functions.

Consequently, we investigated the biological roles of *CLEC12A-MIR223HG* based on the previously reported functions of *CLEC12A*. Previous studies have reported a higher expression of *CLEC12A* in AML cells resistant to cytarabine (AraC) [31]. In contrast to previous observations, the treatment of U-937 cells with AraC did not result in a significant increase in *CLEC12A* expression. Instead, we observed a higher expression of *CLEC12A-MIR223HG*. These discrepancies could be partly explained by the cell line that was used in our study, which is not derived from AML patients. We then overexpressed either *CLEC12A* or *CLEC12A-MIR223HG* in U-937 cells and treated them with AraC. No significant differences in U-937 cell apoptosis were observed in either group. These observations might suggest that the subset of U-937 cells resistant to AraC may express *CLEC12A-MIR223HG* at a slightly higher level, but *CLEC12A-MIR223HG* by itself does not confer resistance to AraC.

Next, we compared the expression levels of the two genes in monocytes and monocytes differentiated into macrophage-like cells. *CLEC12A* is expressed at high levels in granulocyte-monocyte progenitor cells [29] and downregulated in monocytes treated with PMA, which induces their differentiation into macrophage-like cells [45]. We used CD44 and CD45 as markers of differentiation as the expression of CLEC12A is lower in CD45-positive differentiated monocytes [46]. Interestingly, there was a significant increase in the expression of *CLEC12A-MIR223HG*. However, the rate of differentiation of U-937 did not significantly alter following the overexpression of either *CLEC12A* or *CLEC12A-MIR223HG*. Thus, we concluded that the change in expression of *CLEC12A* and *CLEC12A-MIR223HG* could be a marker, but not a significant driver, of monocyte activation and differentiation.

In conclusion, we have identified and characterized a novel chimeric RNA that differs substantially from its parental genes. Our results invite further studies aimed at understanding the roles of *CLEC12A-MIR223HG* and provide an experimental framework to study other chimeric transcripts in normal physiology and cancer. Our results also highlight the need for caution while discovering and reporting novel and potential diagnostic and prognostic cancer biomarkers.

## 4. Materials and Methods

### 4.1. Clinical Samples and Bioinformatics Analysis

Retrieval of patient samples and samples from healthy donors, RNA extraction, library preparation, sequencing, and data analysis has been described previously [28]. In brief, we retrieved 16 diagnostic specimens (total leukocytes from peripheral blood) from treatment naïve CML patients, 10 matched remission samples following successful TKI treatment, and 6 samples from healthy donors. Total RNA was isolated using Trizol and subjected to mRNA sequencing after poly-A-enrichment. Paired-end RNA-sequencing reads (125 nt) were trimmed and mapped to the human reference genome hg38 using STAR v2.7 [47]. STAR-FUSION v1.4.0 [48] was used for the identification of fusion genes and Fusion Inspector (FusionInspector.github.io) for in silico validation of the predicted gene fusions. In addition, we used Arriba [49] to independently reconfirm the *BCR/ABL1* and *CLEC12A/MIR223HG* fusion predictions. GTEx data of 17,382 samples from 30 different tissues were analysed to access correlation between the expression of *CLEC12A* and *MIR223HG*. 

### 4.2. Cell Lines and Culture

HEK293 cells were cultured in Dulbecco’s Modified Eagle Medium (DMEM; Gibco; Waltham, MA, USA, Cat#12430054) supplemented with 10% (*v*/*v*) fetal bovine serum (FBS; HyClone; Marlborough, MA, USA, SH30084.03). Cell lines U-937 (ATCC; CRL-1593.2), K-562 (ATCC; CCL-243) and THP-1 (ATCC; TIB-202) were cultured in Roswell Park Memorial Institute 1640 (RPMI-1640; Gibco; Cat#22400089) medium with 10% FBS. THP-1 cells were additionally supplemented with 1X non-essential amino acids (NEAA; Gibco; Cat#11140050) and 1 mM sodium pyruvate (Gibco; Cat#11360070). HL-60 (ATCC; CCL-240) was cultured in Iscove’s Modified Dulbecco’s Medium (IMDM; Gibco; Cat#12440053) complemented with 20% (*v*/*v*) FBS. DMEM containing 20% (*v*/*v*) FBS was utilised for cell line MOLM-13 (DSMZ; ACC-554) and MEM Alpha (Gibco; Cat#12571063) containing 10% (*v*/*v*) FBS for OCI-AML2 (DSMZ; ACC-99). All growth media contained 100 U/mL penicillin and 100 µg/mL streptomycin (Gibco; Cat#15140122). Cells were maintained in a humidified incubator with 5% CO_2_ at 37 °C. All cell lines used in this study were checked regularly for mycoplasma and authenticated using short tandem repeat profiling.

### 4.3. Expression Vectors, Lentivirus Production, and Transduction

FLAG-CLEC12A and FLAG-CLEC12A-MIR223HG coding sequences were obtained as Geneblocks (IDT Australia) and cloned into a FUW lentiviral plasmid backbone (Addgene #14882) in-frame to an upstream mcherry-P2A sequence. All plasmids were sequence verified with Sanger sequencing (Australian Genome Research Facility). For lentivirus production, HEK293 cells were transfected with packaging plasmids pMD2-VSV-G (Addgene #12259), pRSV-Rev (Addgene #12253), and pMD2-g/pRRE (Addgene #12251) with calcium phosphate. The virus-containing supernatant was collected 48 h post-transfection, filtered through a 0.45 µm filter, and snap-frozen. For transduction, cells (5 × 10^5^) were transferred to FACS tubes (Corning Inc., Corning, NY, USA, Cat#352058). Cells were resuspended in 500 µL of fresh medium and 500 µL of viral supernatant with 4 µg/mL Polybrene (Sigma-Aldrich; St. Louis, MO, USA, Cat#TR-1003). After spinoculation at 1500 rpm for 1.5 h at room temperature, cells were incubated for 4 h at 37 °C, 5% CO_2_. Media was then refreshed, and cells were incubated for 72 h. Transduction efficiency was assessed by measuring the percentage of mCherry^+^ cells by flow cytometry (BD LSRFortessa, Sydney Cytometry) and using FlowJo v10 (BD Biosciences, San Jose, CA, USA) for data analysis.

### 4.4. RNA Isolation and RT-PCR

Total RNA was extracted using TRIzol™ (Thermo Fisher Scientific, Waltham, MA, USA, Cat#15596026) as instructed by the manufacturer. Complementary DNA was subsequently synthesized from total RNA using SuperScript™ III reverse transcriptase (Thermo Fisher Scientific, Cat#18080093) and PCR was performed using a PCR thermal cycler (Eppendorf, Germany, MasterCycler epgradients). The PCR program used for amplification consisted of 2 min at 92 °C, followed by 35 cycles of 5 s at 92 °C, 10 s at 60 °C, and 20 s at 72 °C and concluded with 10 min at 72 °C. Beta-2-Microglobulin (B2M) gene was used to analyse the relative gene expression with either the dCt or the ddCt method. All the primer sequences are listed in Supplementary Table S3.

### 4.5. Western Blotting

Total protein extracts were isolated with NP-40 buffer (1% (*v*/*v*) NP-40, 0.15 M NaCl, 10 mM EDTA, 10 mM NaN_3_, 10 mM Tris-HCl pH 8) containing cOmplete™ Protease Inhibitor Cocktail (Roche, Basel, Switzerland, Cat#116974980001). The protein extracts (20 μg) were separated on SDS PAGE (Bolt™ 4–12% Bis-Tris Plus; Invitrogen; Cat#NW04120BOX) and transferred to Immobilon™-P PVDF membrane (Merck Millipore; Darmstadt, Germany, Cat#IPVH00010). Following the blocking in 5% (*w*/*v*) bovine serum albumin, the membranes were probed with primary antibodies FLAG-HRP (1:1000; Merck; Cat#F1804) or GAPDH mouse monoclonal antibody (1:5000; Abcam; Cambridge, UK, Cat#ab8245) and secondary antibody (1:5000; Merck Millipore; Cat#AP192P). To remove N-linked glycosylation moieties from cell lysates, equal quantities of protein were incubated with PNGase F (New England Biolabs; Ipswich, MA, USA, Cat#P0704S) at 37 °C for 24 h, prior to running on SDS-PAGE gels.

### 4.6. Immunofluorescence

U-937 cells were seeded in ibiTreat µ-Slide 8 Well chambers slide (Ibidi; Germany, Cat#80826) (200 000 cells/well) after being subjected to lentiviral transductions. Cells were fixed with 4% (*w*/*v*) paraformaldehyde (Thermo Fisher Scientific; Cat#28906), permeabilized with Triton X-100 0.2% (Sigma-Aldrich; Cat#X100), and incubated with anti-FLAG mouse monoclonal primary antibody (1:500; Merck; Cat#F1804) and Alexa Fluor 488 rabbit-anti-mouse IgG (1:1000; Invitrogen; Cat# A27023) secondary antibody. Cells were washed with PBS between steps and incubated with DAPI (5 min; 1:5000; Invitrogen; D1306) before mounting with ibidi mounting medium (Ibidi; Cat#50001). Fluorescence images were acquired using a Leica-SP8 confocal microscope with a HC PL APO CS2 40/1.10 water objective and analysed using the Bitplane Imaris software (Imaris, Zurich, Switzerland).

### 4.7. Liquid Chromatography with Tandem Mass Spectrometry (LC-MS/MS) Analysis

In order to map the protein interaction profile of CLEC12A and CLEC12A-MIR223HG, FLAG-tagged protein pulldown followed by label-free quantification was performed. Whole cell extracts of U-937 cells expressing FLAG-CLEC12A and FLAG-CLEC12A-MIR223HG were incubated with 30 μL anti-FLAG beads (Sigma-Aldrich) for 2 hr. Six washes using a buffer containing 200 mM NaCl, 50 mM Tris-HCl, 0.25% (*v*/*v*) IGEPAL, pH 7.9 were performed (5 min each; rotating end-to-end). Affinity-purified protein complexes were subjected to on-bead trypsin/LysC digestion in 2 M urea for 1 h at 30 °C. Next, the beads were collected and the supernatant was transferred into low retention tubes. Beads were resuspended in 25 μL of 2 M urea containing 20 mM IAA for 20 min. Tryptic peptides were acidified to a final concentration of 2% (*v*/*v*) with formic acid (Sigma Aldrich) and desalted using ZipTips (Thermo Fisher Scientific). Liquid Chromatography with tandem mass spectrometry (LC-MS/MS) analysis was performed on a Thermo Scientific Q-Exactive HF-X hybrid quadrupole-Orbitrap mass spectrometer. Raw data were analysed by MaxQuant (version 1.6.6.0) [50] using standard settings. Methionine oxidation (M) and carbamidomethyl cysteine (C) were selected as variable and fixed modifications, respectively. Identified peptides were searched against the reference human proteome. Proteins with less than 2 unique peptides and more than one LFQ missing values were omitted from the analysis. Perseus algorithm was used to impute the missing values [51]. Output files were further processed and keratin, heat shock, and ribosomal proteins were excluded from the analysis. To detect fusion-specific unique peptides, we allowed up to four missed-cleavage and selected match between runs and dependent peptides.

### 4.8. CRISPR-SAM-Mediated Activation of CLEC12A and Analysis of Chromosomal Translocations in HEK293 Cells

HEK293 cells were transduced with dCas9-VP64_Blast (Addgene #61425) and MS2-p65-HSF1_Hygro (Addgene #61426) lentiviruses. The media was replenished with 2 µg/mL blasticidin (AG Scientific, CA, USA, Cat# B-1247) and 400 µg/mL hygromycin (Roche, Cat#10843555001) 24 hrs post-transduction. The cells were selected for 7 days and the surviving cells were referred to as HEK293-dCas9/MPH. The sgRNA to activate *CLEC12A* was designed as previously described [52]. SgRNA was obtained as single-stranded oligonucleotides (IDT Australia), treated with T4 Polynucleotide Kinase (NEB #M0201), annealed and ligated to lenti sgRNA(MS2)_puro (Addgene #7379) digested with *BsmBI* (NEB, Cat #R0580). A 20 bp non-targeting scrambled sequence in the plasmid backbone was used as a non-targeting control. The plasmids were verified by Sanger Sequencing (Australian Genome Research Facility). For the activation of *CLEC12A*, HEK293-dCas9/MPH cells were transduced with lenti sgRNA(MS2) lentiviruses expressing CLEC12A-targeting sgRNA (SAM-CLEC12A-sgRNA) by spinoculation in a 12-well plate and selected with 2 µg/mL puromycin (Gibco, Cat#A1113803). The expression of *CLEC12A* and *CLEC12A-MIR223HG* was then quantified with qRT-PCR. Whole genome sequencing data for HEK293 were obtained from NCBI SRA (SRR2123657). Raw fastq files were downloaded from ENA and aligned to the hg38 using Speedseq tool that internally uses BWA-MEM for alignment (https://github.com/hall-lab/speedseq#speedseq-realign) (accessed on 26 October 2021) [53].

### 4.9. U-937 Monocyte Differentiation Assay

In our method, 10^6^ U-937 monocytic cells were differentiated with 100 nM PMA (phorbol 12-myristate 13-acetate) (Sigma, Cat#P8139) in 6-well plates. Differentiated cells were collected using the StemPro™ Accutase™ Cell Dissociation Reagent (Life Tech, Cat#A1110501) 48 h post-treatment. Collected cells were washed with PBS and either processed for flow cytometry or RNA extraction. For flow cytometry, cells were washed twice with PBS and stained with CD44-PE (BD, cat# 555479) and CD45-APC (BD Biosciences, cat#559864) for one hour in the dark. Following incubation, the cells were washed twice with PBS and analysed with flow cytometry (BD LSR Fortessa, Sydney Cytometry) and data visualised using FlowJo v10 (BD Biosciences).

### 4.10. Cell Apoptosis with Annexin V Staining

U-937 cells were seeded in 6-well plates (500,000 cells/well/replicate) and incubated with Cytarabine (AraC, Sigma-Aldrich; Cat#147-94-4) for 72 h. The cells were collected, washed with PBS, and incubated for 15 min in the dark with Annexin V-APC (BD Biosciences; Cat#550474) and DAPI (1:1000). The percentage of apoptotic cells was quantified with flow cytometry and data visualised using FlowJo v10 software.

### 4.11. Cell Viability Assay

MTT Formazan powder (Sigma-Aldrich; Cat#88417) was dissolved in sterile PBS at a working concentration of 5 mg/mL. The solution was then filtered using a 0.22 µm syringe filter and stored at 4 °C for short-term and −20 °C for long-term use. U-937 cells were seeded in 96-well plates (2000 cells/well/replicate) after lentiviral transductions. Cells were then incubated with AraC for 72 hr. MTT solution was added at 0.5 mg/mL and incubated overnight prior to the addition of 110 µL of the Colour Development Solution (isopropanol with 0.04 N HCl). Absorbance was recorded with the POLARstar microplate reader (BMG LABTECH; VIC, AUS) at 570 and 630 nm wavelength. Calculations were performed using the equation A_570nm_–A_630nm_. Media only control wells were then subtracted from all the readings. The addition of MTT solution and colour development solution was repeated at 0 and 72 h, with plate absorbance readings occurring at the same time point.

### 4.12. Colony Formation-Methocult Assay

U-937 cells were diluted in media at a concentration of 100 cells/µL. IMDM + 2% FBS (900 µL) (StemCell Technologies; Vancouver, Canada, Cat#07700) was then added to the cells and mixed thoroughly. Cell mixture (1 mL) was added to 3 mL of Methocult Media H4230 (StemCell Technologies; Cat#04230). The methocult/cell mixture (1 mL) was then aliquoted into gridded 35 mm tissue culture dishes in triplicate (Sarsedt; Germany, Cat#83.3900.002). Dishes were incubated 5% CO_2_ at 37 °C for 8 days. Colony numbers were counted and analysed with GraphPad Prism 8.

### 4.13. Statistical Analysis

All data are reported as mean ± SD of at least three independent experiments. Data with two groups to compare were analysed using unpaired, two-tailed *t*-test in GraphPad Prism 8. Pearson correlation coefficient was used to access the correlation between the expression of *CLEC12A* and *MIR223HG*. The significance level chosen for the statistical analysis were ** *p* < 0.01, * *p* < 0.05.

## Data Availability

RNA sequencing data can be accessed at Gene Expression Omnibus (GEO, https://www.ncbi.nlm.nih.gov/geo/) accession number: GSE144119.

## Supplementary Materials

The following are available online at https://www.mdpi.com/article/10.3390/ijms222212178/s1, Supplementary Figure S1: Scatterplots illustrating correlation between *CLEC12A* and *MIR223HG* in individual tissues. Figure S2, A: Detection of lentivirus-mediated overexpression of *CLEC12A* and *CLEC12A-MIR223HG* in different cell lines, B: cell growth and proliferation assays after overexpression of *CLEC12A* and *CLEC12A-MIR223HG*, C: Examination of U-937 apoptosis after cytarabine treatment at different doses. D: Percentage of apoptotic U-937 cells after cytarabine treatment. E: PCR on the genomic DNA and cDNA of U-937 cells to detect *CLEC12A-MIR223HG*. Supplementary Table S1: List of fusion transcripts involving *CLEC12A* in CML patients, Supplementary Table S2: Cellular localization of interactors of CLEC12A and CLEC12A-MIR223HG identified with pull down/mass spectrometry, Supplementary Table S3: List of all the primers used in this study, Supplementary Table S4: List of all chromosomal translocations in HEK293 cells.
