# Supplementary material for: The Fusion of CLEC12A and MIR223HG Arises from a trans-Splicing Event in Normal and Transformed Human Cells
Source: Int J Mol Sci. 2021 Nov 10;22(22):12178. doi: 10.3390/ijms222212178 (PMC8625150; doi:10.3390/ijms222212178)
Supplement: Supplementary file 1 [file ijms-22-12178-s001.zip › ijms-1414506-supplementary.pdf]

Supplementary Table S1: A list of fusion events involving *CLEC12A* in the PBMCs of CML patients.

Left Gene	Left break point	Right break point	Right gene
CLEC12A	12:9951356:+	7:770471:+	*DNAAF5*
CLEC12A	12:9982129:+	21:33567157:+	*SON*
CLEC12A	12:9982129:+	X:66020127:+	*MIR223HG*
CLEC12A	12:9982129:+	1:185305643:-	*IVNS1ABP*
CLEC12A	12:9982129:+	16:89647331:-	*CHMP1A*
CLEC12A	12:9982129:+	22:36305102:-	*MYH9*
CLEC12A	12:9982129:+	6:35869901:-	*SRPK1*
CLEC12A	12:9982129:+	1:1390865:-	*CCNL2*
CTD	14:38194142:-	12:9971577:+	*CLEC12A*

Supplementary Table S2: Cellular localization of interactors of CLEC12A and CLEC12A-MIR223HG identified with pull down/mass spectrometry (based on Uniprot).

Protein	Interactor	Localization
CALX	CLEC12A-MIR223HG	Endoplasmic reticulum membrane
XPO5	CLEC12A	Cytoplasm
RHG04	CLEC12A-MIR223HG	Cytoplasm
PSA5	CLEC12A/CLEC12A-MIR223HG	Cytoplasm
A2MG	CLEC12A-MIR223HG	Secreted
RCN2	CLEC12A-MIR223HG	Endoplasmic reticulum lumen
RASL3	CLEC12A-MIR223HG	Cytoplasm
LUC7L	CLEC12A-MIR223HG	Nucleus

Supplementary Table S3: A list of primers used in this study.

Primer name	Primer sequence
CLEC12A-MIR223HG-Fwd/ Primer A/ qPCR primer	GGCTGGGATTATCTCCTGAAG
CLEC12A-MIR223HG-Rev/ Primer B/ qPCR primer	TTAGGCAGATGGCTGGTTG
CLEC12A-Rev/ qPCR primer	TGTTATTTAAGTCAGGTGCGTTTC
MIR223HG-F	AATGAGGCATTTGCCATACC
MIR223HG -R	TGAAAAGAGGAAAAGATCTCCA
hB2M-F/ qPCR primer	TGCCGTGTGAACCATGTGAC
hB2M-R/ qPCR primer	CGGCATCTTCAAACCTCCAT
Primer C	AGTGCATATATTTATTGCAGCATCTGGATC
Primer D	TGAACTCCTCAGTGCATATATTTATTGCAGC
Primer E	GTCAGGCCTGTGAACTCCTCAG
Primer F	GAAAGGAGACCAAAAGTCAGGCCTG
Primer G	GGCTCATTTGCAGACATATGGGTGATTGGTAC
Primer H	GAGAAGAAATGTACTATTCCCAACA
Primer I	TTGGGGTTTGGGAAATTAGC
SAM-CLEC12A	CACTCACTAATCTGCAGCTG

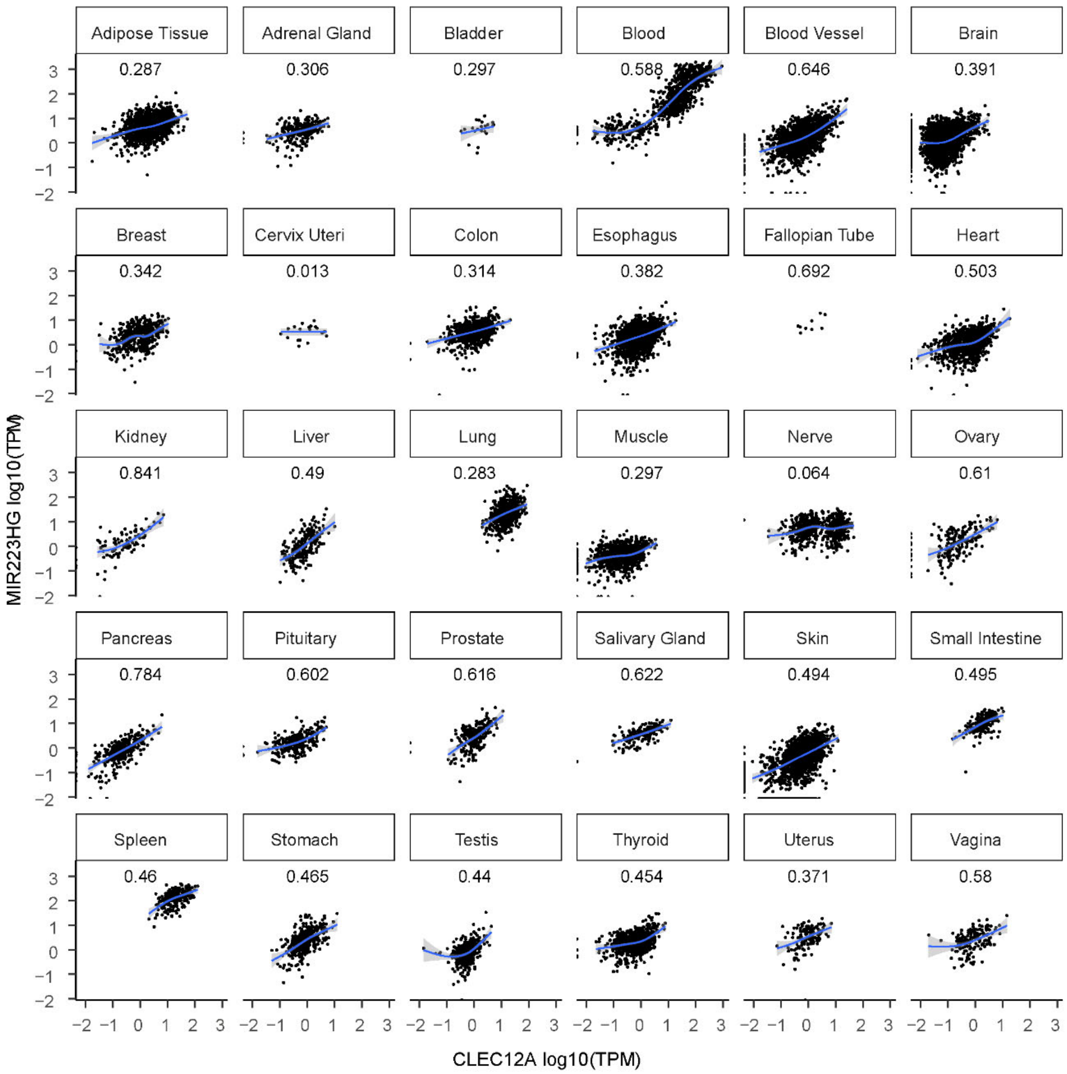

Figure S1: Scatterplots illustrating correlation between *CLEC12A* and *MIR223HG* expression in individual tissues. Transcripts per million (TPM) values were received from the GTEx Portal (V8, gtexportal.org). Correlation coefficient was determined using Pearson correlation.

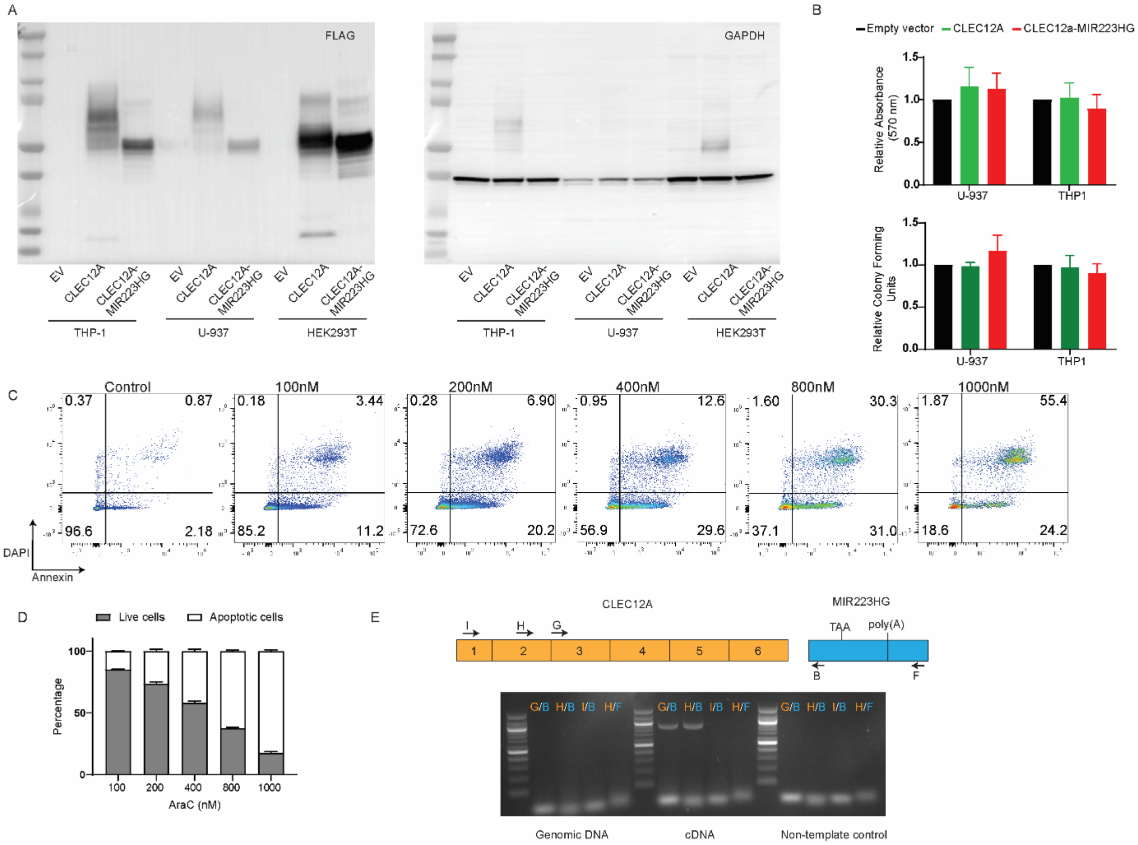

Figure S2 (A) Western blot analysis of lentivirus-mediated overexpression of *CLEC12A* and *CLEC12A-MIR223HG* in THP1, U-937 and HEK293T cell lines. **(B)** MTT and clonogenicity assays on THP1 and U-937 cell lines overexpressing either *CLEC12A* or *CLEC12A-MIR223HG*. **(C)** Dose-dependent increase in the apoptosis of U-937 cells treated with cytarabine (AraC). Representative flow plots are shown at different cytarabine (AraC) doses. **(D)** Percentage of apoptotic U-937 cells treated with different concentrations of AraC. **(E)** PCR on the genomic DNA and cDNA of U-937 cells to detect *CLEC12A-MIR223HG*. Expected amplicons sizes are as follows: G/B: 962 bp and H/B: 829 bp.
